# The role of lncRNA SNHG15 in UV-induced DNA damage repair

**DOI:** 10.1371/journal.pone.0334414

**Published:** 2025-10-09

**Authors:** Xin Zhang, Jiahui Lin, Zixuan Fan, Sai Zhang, Meiqi Yang, Tingting Zhu, Qing Lu, Yuming Wang

**Affiliations:** 1 Department of Neurology, Institute of Neuroscience, Key Laboratory of Neurogenetics and Channelopathies of Guangdong Province and the Ministry of Education of China, The Second Affiliated Hospital, Guangzhou Medical University, Guangzhou, China; 2 Department of Neurology, Guangzhou First People’s Hospital, Guangzhou Medical University, Guangzhou, China; Dr Vishwanath Karad MIT World Peace University, INDIA

## Abstract

Aberrant expression of long noncoding RNAs (lncRNAs) serves as a critical driver in carcinogenesis and tumor evolution across various human cancers. Among these lncRNAs, small nucleolar RNA host gene 15 (*SNHG15)* has emerged as a key regulator in multiple cellular processes. Its dysregulation is frequently implicated in disease pathogenesis, particularly cancer. In this investigation, we delineated the functional landscape and mechanistic basis of *SNHG15* in lung carcinoma. Cell cycle synchronization experiments demonstrated phase-dependent *SNHG15* expression, showing maximal transcript abundance during G2/M transition. *SNHG15* knockdown substantially curbed tumor growth in vitro (A549 cell line) and in vivo (subcutaneous PDX models). Notably, *SNHG15* orchestrated genomic surveillance mechanisms through tripartite regulation: (1) cell cycle checkpoint coordination, (2) transcriptional machinery reactivation, and (3) nucleotide excision repair. Mechanistically, proteomic profiling coupled with RNA immunoprecipitation identified CREB5 as a direct binding partner mediating *SNHG15*-dependent survival signaling under genotoxic stress. These results position the *SNHG15*-CREB5 axis as an important player governing genomic fidelity in lung adenocarcinoma, suggesting druggable targets for precision oncology approaches.

## Introduction

Emerging insights from pan-cancer genomic investigations have revealed extensive dysregulation of long non-coding RNAs (lncRNAs) across malignancies. Large-scale tumor omics studies demonstrate that transcriptional abnormalities and somatic variations in these RNA molecules significantly contribute to oncogenic transformation, metastatic progression, and therapeutic resistance across multiple cancer models [1–3]. Despite their emerging diagnostic potential and therapeutic relevance, functional characterization remains limited for most lncRNAs due to their complex regulatory network and context-dependent activities.

Notable among cancer-associated lncRNAs is HOX transcript antisense RNA (*HOTAIR*), initially characterized through comparative expression profiling in metastatic breast carcinomas. Clinical evidence correlates elevated *HOTAIR* levels with aggressive tumor phenotypes and diminished patient survival [4,5]. Mechanistically, this lncRNA facilitates chromatin remodeling through spatial recruitment of EZH2-containing PRC2 complexes, enabling epigenetic silencing of metastasis suppressor genes [5,6]. The metastasis-associated lung adenocarcinoma transcript 1 (*MALAT1*) exemplifies another oncogenic lncRNA with conserved structural features. Following post-transcriptional maturation, its stable nuclear isoform modulates pre-mRNA processing through interactions with serine/arginine-rich splicing factors. Functional studies reveal *MALAT1*’s dual regulatory capacity: acting as molecular sponge for tumor-suppressive miRNAs through competitive endogenous RNA mechanisms, and facilitating epithelial-mesenchymal transition via extracellular matrix remodeling pathways [7,8]. X-inactive specific transcript (*XIST*), the paradigm-setting lncRNA essential for X-chromosome dosage compensation, exhibits paradoxical oncogenic activation in various malignancies. Beyond its canonical role in chromatin silencing, accumulating evidence suggests *XIST* participates in post-transcriptional regulation of oncogenic signaling cascades through protein complex scaffolding [9,10].

Emerging evidence highlights the oncogenic versatility of the small nucleolar RNA host gene (*SNHG*) long non-coding RNA family across neoplastic diseases [11–13]. Initially characterized in gastric carcinoma with elevated expression patterns, *SNHG15* has subsequently been validated as a clinically relevant indicator for hepatic carcinoma outcomes [14]. Previous studies demonstrate that altered *SNHG15* levels exhibit strong associations with core tumorigenic processes including cellular replication, apoptosis, tissue invasion, and metastatic dissemination in human malignancies [15]. Notably, this molecule has been implicated in therapeutic resistance mechanisms observed in pulmonary adenocarcinoma [16]. The multifaced involvement of *SNHG15* in oncogenic regulation and clinical manifestations positions it as a molecular player in cancer progression. Recent mechanistic investigations have substantially advanced our comprehension of *SNHG15*’s functional spectrum in both normal physiology and disease states, reinforcing its potential utility in clinical oncology [17–19].However, methodological discrepancies across research groups studying long non-coding RNAs, compounded by inherent biological complexities and tumor diversity, have occasionally yielded conflicting observations. Resolving the molecular circuitry governing *SNHG15*-mediated neoplastic transformation remains critical for establishing unified pathogenic models.

In this study, we identified *SNHG15* transcriptional activation following genotoxic stress, with peak expression coinciding with G2 phase progression. Depletion of *SNHG15* induced cell cycle blockade at G0/G1 transition points. Functional depletion significantly compromised nucleotide excision repair capacity in malignant cells and heightened therapeutic susceptibility. Proteomic analysis uncovered cooperative interactions between *SNHG15* and CREB5, a transcriptional regulator responsive to cAMP signaling, mediating these biological effects. These findings expand the mechanistic repertoire of long non-coding RNAs by revealing *SNHG15*’s regulatory functions in ultraviolet-induced DNA damage responses through post-transcriptional coordination.

## Results

### Induction of *SNHG15* after DNA damage

**Fig 1:**
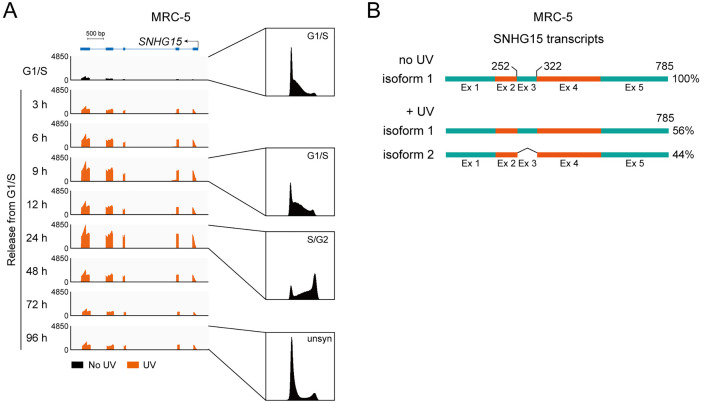
Induction of *SNHG15* upon DNA damage. (A) This panel presents an Integrative Genomics Viewer (IGV) snapshot displaying RNA-sequencing (RNA-seq) data tracks corresponding to the lncRNA *SNHG15* in human lung fibroblast MRC-5 cells. MRC-5 cells were arrested at the G1/S phase boundary using double thymidine block to ensure uniform cell cycle progression. After synchronization, cells were released into the cell cycle and subsequently exposed to 10 J/m^2^ UV-C irradiation. Total RNA was extracted post-treatment, followed by library preparation and sequencing, and alignment to the human reference genome (GRCh38). Top schematic, a linear diagram of the *SNHG15* locus with exons (boxes) and introns (lines). Scale bar, indicating genomic coordinates (bp). Flow cytometry analysis of cell cycle distribution at specified time points post-release was presented. (B) Schematic representation depicting the distinct full-length transcript variants of the lncRNA *SNHG15*, elucidated through 5’ and 3’ RACE analysis performed in MRC-5 cells. See also S1 Fig.

To explore the potential biological significance of *SNHG15* under genotoxic stress, we performed systematic expression profiling of non-coding transcripts in UV-C irradiated human lung fibroblasts MRC-5 during a 96-hour recovery phase (unpublished data). Transcriptome-wide analysis revealed broad suppression of lncRNA biogenesis, consistent with DNA damage-induced transcriptional arrest. Intriguingly, *SNHG15* displayed an immediate induction profile, peaking specifically at 24 hours post-irradiation (Fig 1A). This temporally restricted activation implies functional specialization in genomic stress adaptation. RefSeq annotations indicate transcript heterogeneity for *SNHG15*, which we experimentally confirmed through RACE analysis identifying two predominant isoforms (785 and 715 nt). UV exposure selectively upregulated the shorter 715-nt variant, demonstrating stimulus-dependent splicing regulation (Fig 1B, S1 File). Accordingly, in A549 human lung carcinoma cells, UV irradiation also specifically upregulated the expression of isoform 2 of *SNHG15* (S1A Fig). Neither of the two transcript isoforms of *SNHG15* exhibited significant upregulation or downregulation in tumor cells when compared with their expression levels in normal tissue cells (S1B Fig). *In vitro* transcriptional/translational assays excluded protein-coding potential for both variants (S1C Fig).

Not all DNA damaging agents equally modulated *SNHG15* expression: cisplatin (induces intrastrand crosslinks) caused moderate upregulation, whereas 5-FU (thymidylate synthase inhibitor) and doxorubicin (topoisomerase II poison) showed no significant effect in A549 cells (S1D Fig). This agent-specific response pattern dissociates *SNHG15* from classical double-strand break repair pathways (HR/NHEJ), suggesting preferential involvement in nucleotide excision repair or replication stress response mechanisms. Furthermore, testing across multiple normal and cancerous cell lines consistently revealed that UV-C irradiation robustly upregulates *SNHG15* expression regardless of cellular origin (S1E Fig), underscoring its role as a stress-responsive lncRNA that modulates DNA damage repair pathways and cell survival.

### *SNHG15* loss of function results in cell cycle arrest

**Fig 2:**
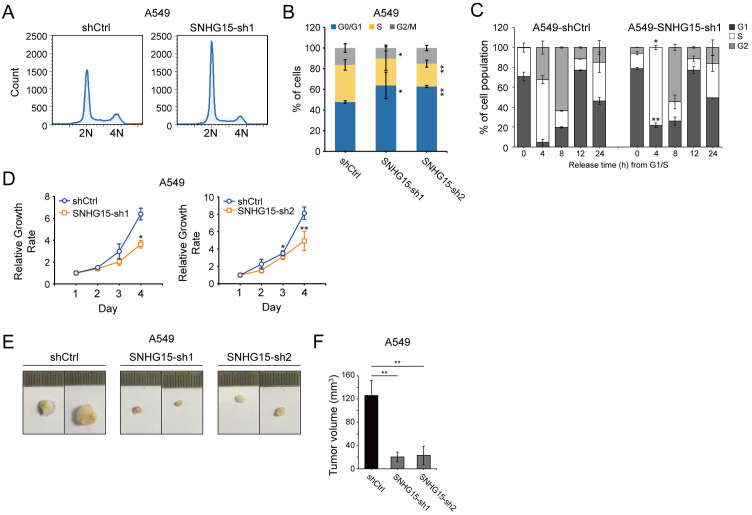
*SNHG15* knockdown reduces cell proliferation and regulates the cell cycle. (A) Cell cycle profiles of *SNHG15*-depleted A549 cells. The KD cells exhibited a pronounced increase in the proportion of cells residing in the G1 phase. (B) Cell cycle distribution. Quantitative analysis of cell cycle progression in wild-type and *SNHG15*-depleted A549 cells revealed significant perturbations upon *SNHG15* knockdown. All bars represent means ± S.D. from three biologically independent experiments normalized to account for potential plate-to-plate variability. Statistical analysis for differences in cell populations in each phase compared to control. *p < 0.05, and **p < 0.01 (Student’s *t*-Test). (C) Quantitative analysis of cell cycle phase distribution in wild-type and *SNHG15*-knockdown A549 cells at specific time points following G1/S phase synchronization release. All bars represent mean percentages ± S.D. derived from three independent biological replicates. Statistical significance of phase population differences relative to control groups was determined by two-tailed Student’s t-test. *p < 0.05, and **p < 0.01. (D) Relative proliferation rates were determined using MTT proliferation assay in A549 cells. Results were normalized to day 1. Mean values of three independent experiments ± S.D are presented. Statistical analysis for differences in cell proliferation rates in *SNHG15*-deficient cells compared to WT. *p < 0.05, and **p < 0.01 (Student’s *t*-Test). (E) Representative images illustrating *t*he gross morphology of xenograft tumors excised from immunodeficient nude mice following subcutaneous implantation of human A549 cells. Visual comparison clearly demonstrates that tumors derived from *SNHG15*-KD cells exhibit a substantially reduced size compared to those derived from WT cells. (F) Quantification of the tumor volumes in xenograft models. Tumor volumes were measured longitudinally and presented as means ± S.D. (n = 12 mice per group). A statistically significant reduction in tumor volume was observed in the shSNHG15 group compared to the shCtrl group (**p < 0.01, two-tailed Student’s t-test), indicating a potential inhibitory role of *SNHG15* knockdown in tumor progression. See also S2 and S3 Figs.

To investigate *SNHG15*’s involvement in cell division dynamics, we synchronized human fibroblast cells (MRC-5) at the G1/S boundary and monitored transcriptional fluctuations across mitotic phases. Transcriptome profiling uncovered periodic *SNHG15* activity, displaying minimal output during early division phases followed by peak expression in the post-replicative phase (S2A Fig). This cyclical transcriptional behavior was replicated in pulmonary adenocarcinoma A549 cells (S2B Fig). To establish functional correlations between *SNHG15*’s periodic expression and mitotic regulation, we disrupted *SNHG15* expression in A549 cells and H1299 cells using dual exon-targeting RNA interference constructs designed against alternative transcript variants. Quantitative reverse transcription PCR validated successful suppression of both transcript isoforms (S2C Fig). Flow cytometry analysis demonstrated altered phase distribution in gene-silenced populations, with notable G1 accumulation and subtle reductions in DNA synthesis and division phases in both A549 and H1299 cells (Figs 2A-B and S3D). For temporal resolution of cell cycle perturbations, we implemented sequential thymidine blockade to generate phase-homogeneous populations, maintaining synchronization fidelity while allowing natural progression post-release. In parental A549 cells, approximately 30% of the population entered post-synthetic phase within 4 hours of blockade removal (Fig 2C). Strikingly, *SNHG15*-deficient counterparts displayed complete absence of post-replicative cells at matching timepoints, indicating impaired S-phase transition and delayed G2 entry following *SNHG15* suppression.

Building upon these findings, we investigated *SNHG15*’s functional contributions to autonomous tumor growth under basal conditions. Comparative analysis of baseline proliferative capacity between parental and *SNHG15*-silenced lung cancer cell lines revealed quantitative disparity in metabolic activity measurements (Fig 2D and S2E). Additionally, *SNHG15*-depleted A549 cells displayed an obvious growth delay in clonogenic assays (S2F Fig). In translational modeling, immunocompromised murine models receiving *SNHG15*-deficient xenografts developed substantially diminished neoplastic masses compared to control samples (Figs 2E-F and S2G). The perturbation of *SNHG15* expression additionally attenuated cellular motility, as evidenced by impaired wound closure dynamics in lung cancer cells (S2H-I Fig). *SNHG15* KD A549 cells exhibited complete would closure failure at 72 hours (S2J Fig). These results support *SNHG15*’s dual regulatory function in both mitotic control and malignant expansion within pulmonary neoplasms.

While histone H3 serine 10 phosphorylation (p-H3S10) exhibits temporal coordination with mitotic progression [20] and functions as an oncogenic facilitator through malignant reprogramming mechanisms [21], our interrogation of *SNHG15*-depleted A549 cell populations revealed preserved phosphorylation dynamics across cell cycle phases (S3A Fig). Intriguingly, this experiment unexpectedly identified a significant reduction in micronuclear frequency following *SNHG15* suppression (S3B Fig). Given micronuclei’s established association with chromosomal instability and tumorigenic evolution [22], these data position *SNHG15* as a novel epigenetic modulator of nuclear integrity during carcinogenic progression.

### Knockdown of *SNHG15* impairs nucleotide excision repair in lung cancer cells

**Fig 3:**
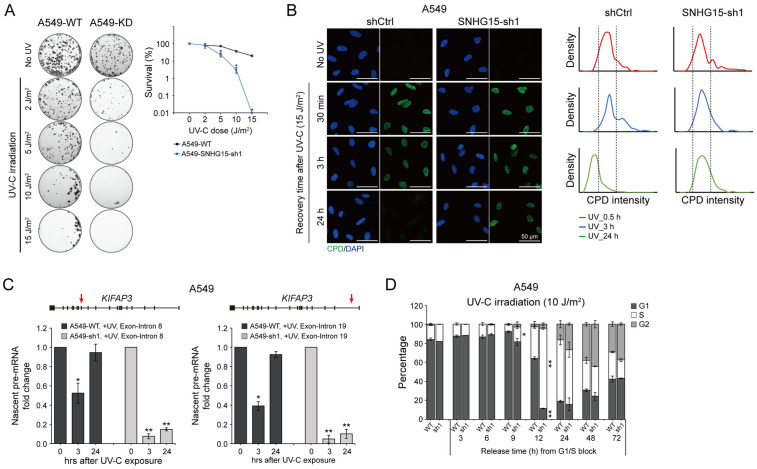
*SNHG15* facilitates TC-NER in response to DNA damage. (A) (*left panel*) representative images of clonogenic survival assay showing *SNHG15*-depleted A549 cells exposed to varying UV-C doses. (*right panel*) Survival rates (logarithmic scale) as a function of UV-C dose, with error bars representing standard deviation from three independent biological replicates. *p < 0.05 versus shCtrl controls (Student’s t-test). (B) Representative immunofluorescence images and quantitative analysis of CPD repair kinetics in wild-type and *SNHG15*-depleted A549 cells. Cells were stained with anti-CPD antibody (green) at specified time points post-UV irradiation, with DAPI counterstaining (blue) indicating nuclei. Scale bar = 50 μm. (C) Nascent mRNA production in different regions of the human *KIFAP3* gene. A549 cells were irradiated with 15 J/m^2^ UV-C. Error bars represent means ± SD from three independent experiments. *p < 0.05 compared to the shCtrl cells (Student’s t-test). (D) Flow cytometry analysis of cell cycle progression in wild-type and *SNHG15*-depleted A549 cells after UV-C irradiation. The bar charts show the percentage of cells at different cell cycle phases at specified time points following release from G1/S synchronization. Each data point is presented as means ± SD from three independent experiments. *p < 0.05, **p < 0.01 compared to wild-type controls at each time point (Student’s t-test). See also S4 Fig.

To explore *SNHG15*’s functional involvement in DNA damage response mechanisms, we implemented lentiviral delivery of short hairpin RNA optimized for maximal silencing efficacy in lung carcinoma models. Due to the higher knockdown efficiency exhibited by shRNA1 construct compared to shRNA2, we primarily employed shRNA1 to achieve target gene depletion in subsequent functional experiments. Silencing of *SNHG15* in A549 cells significantly enhanced cellular vulnerability to UV-C induced genotoxic stress (Fig 3A). Importantly, gene expression profiling of *SNHG15* upon UV-C irradiation and colony formation results in H1299 cells consistently support the findings in A549 cells (S4A-B Fig).

Through comprehensive genomic analysis of DNA repair dynamics, we observed distinct temporal patterns between two major UV-induced photolesions: [6–4] pyrimidine-pyrimidone adducts demonstrated rapid resolution, whereas cyclobutane pyrimidine dimers (CPDs) – both generated through UV-C exposure – exhibited protracted clearance kinetics through nucleotide excision repair (NER) pathways [23]. Given their characteristic 12–48 hour resolution window through NER mechanisms, CPDs provide more reliable biomarkers for evaluating transcription-coupled NER (TC-NER) capacity. Notably, *SNHG15*-deficient lung cancer cells displayed impaired CPD resolution kinetics, with persistent lesions detectable at 24-hour post-irradiation (Fig 3B and S4C).

Cells deficient in specific DNA repair factor genes fail to restore RNA synthesis from damaged genomic loci and exhibit impaired RNAPII-mediated transcriptional elongation post-UV exposure, whereas wild-type counterparts regain transcriptional activity within hours after irradiation [24,25]. To investigate *SNHG15*’s potential role in coordinating genome-wide transcriptional responses to UV damage, we established an experimental system using both A549 and H1299 cells: cells were pretreated with the transcription elongation inhibitor DRB to reversibly arrest newly initiated RNAPII complexes, followed by UV irradiation and subsequent inhibitor removal. This synchronization protocol enabled precise assessment of nascent transcript progression across various intron-exon boundaries. The result (Figs 3C and S4D) shows that UV-C exposure comparably suppressed RNAPII progression in both wild-type and *SNHG15*-depleted cells. Notably, while arrested pre-mRNA levels at both the first and last exon-intron junctions fully recovered in wild-type cells by 24 hours post-UV exposure, this transcriptional restoration remained absent in *SNHG15*-depleted cells.

To investigate the functional necessity of *SNHG15* in DNA damage-induced cell cycle control, we established a synchronized progression model using A549 cells: G1/S phase-arrested populations were subjected to UV-C exposure before S-phase release. Quantitative cell cycle profiling revealed an unexpected kinetic shift – compared to wild-type counterparts, *SNHG15*-deficient populations displayed accelerated G1 phase exit and premature S/G2 phase accumulation within the 9–12 hour post-release window (Fig 3D). This aberrant progression pattern suggests compromised checkpoint enforcement following *SNHG15* depletion, potentially undermining damage surveillance prior to replication. Mechanistically, these observations position *SNHG15* as a critical modulator of lesion-specific repair processes governing genomic integrity maintenance in pulmonary malignancies.

### *SNHG15* promotes DNA damage repair by targeting CREB5

**Fig 4:**
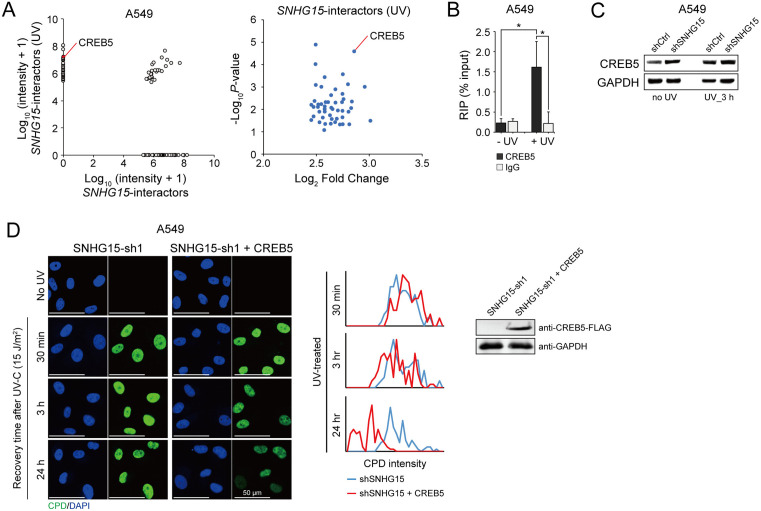
Identification of UV-induced *SNHG15* interactors. (A) The UV-induced interactome of *SNHG15* was characterized using RNA pull-down experiments, where biotinylated *SNHG15* transcripts were incubated with extracts from A549 lung adenocarcinoma cells exposed to UV-C irradiation. Proteins specifically bound to *SNHG15* under genotoxic stress conditions were subsequently isolated via streptavidin affinity capture and identified by mass spectrometry. (B) RNA immunoprecipitation (RIP) targeting CREB5 in A549 cells demonstrated significant enrichment of *SNHG15* RNA, as assessed by RT-PCR. * *P* < 0.05 compared to untreated and IgG controls (Student’s t-test). Means ± SD are shown, *n* = 3 independent experiments. (C) Immunoblot analysis was performed to examine the expression levels of CREB5 protein in A549 cells under UV irradiation. Two experimental groups were established: control cells transfected with scramble shRNA (shCtrl) and experimental cells transfected with *SNHG15*-targeting shRNA (shSNHG15). (D) (*left panel)* representative immunofluorescence images showing CPD repair in A549 cells at indicated recovery times post-UV irradiation. Images depict SNHG15-depleted cells and SNHG15-knocked down cells overexpressing CREB5, stained with a CPD-specific antibody (green) and DAPI (blue). Scale bar = 50 μm. (*right panel)* histogram plots showing average CPD signal intensity at indicated recovery time points following UV-C irradiation. Western blot confirms overexpression of CREB5 in *SNHG15*-depleted A549 cells.

To clarify the molecular basis of *SNHG15*’s involvement in DNA damage response pathways within pulmonary carcinoma models, we employed affinity capture techniques using biotin-tagged *SNHG15* probes followed by proteomic profiling. Our experimental data indicated that this lncRNA establishes distinct protein interaction networks under basal conditions versus UV-C stimulated situations in A549 cells, with quantitative analysis identifying 50 irradiation-responsive binding partners showing substantial enrichment (Fig 4A). Subsequent ribonucleoprotein immunoprecipitation studies demonstrated UV activation-dependent association between *SNHG15* and CREB5 (Fig 4B). Notably, suppression of *SNHG15* showed no regulatory impact on CREB5 protein levels (Fig 4C). Restoration experiments revealed that ectopic CREB5 expression effectively ameliorated DDR functional impairments in *SNHG15*-deficient A549 cells. Particularly, CREB5 reconstitution in *SNHG15*-silenced systems significantly enhanced the clearance rate of CPD lesions following UV irradiation (Fig 4D).

## Discussion

Accurate identification of neoplastic conditions at initial stages demonstrates critical importance in extending clinical outcomes for oncology cases. Current investigations have established that circulating long non-coding RNA molecules exhibit substantial potential as reliable diagnostic indicators for tumor identification [26,27]. While thyroid malignancies display an exception pattern [28], elevated *SNHG15* expression manifests broadly across various carcinomas and participates in oncogenic mechanisms through multiple validated molecular cascades [29]. Our findings identified predominant accumulation of this lncRNA during G2 phase in pulmonary carcinoma cultures. Suppression of *SNHG15* triggered cell cycle arrest at G1/S transition phase, thereby inhibiting cell proliferation *in vitro*. Although existing reports present divergent mechanistic interpretations regarding its pro-proliferative functions [30,31], both our evidence and parallel studies confirm *SNHG15*’s regulatory impact on mitotic progression. Notably, our studies uncovered reduced micronucleus formation in genetically modified A549 clones with *SNHG15* depletion, suggesting this molecule may potentiate chromosomal instability in lung cancer cells. Chromosomal fragility represents a fundamental oncological characteristic enabling acquisition of tumorigenic capacities, commonly attributed to defects in DNA repair mechanisms. Our data illustrating *SNHG15*’s involvement in genomic maintenance processes supports a hypothetical model where its overexpression could induce defective DNA restoration, subsequently accumulating mutagenic changes that accelerate neoplastic transformation. *SNHG15*’s proliferative role aligns with prior reports in gastric [32] and colorectal cancers [33], but we newly establish this mechanism in lung adenocarcinoma. This consistency across malignancies suggests *SNHG15* may be a pan-cancer regulator. Our assessment of mitotic activity following *SNHG15* knockdown relied solely on quantification of cells positive for phosphorylation of histone H3 at serine 10 (pH3S10), a well-established marker for chromatin condensation during late G2 and mitosis. While this provides a specific and quantitative measure of cells actively entering or undergoing division, it represents a single methodological approach. Future studies employing complementary techniques such as additional mitotic markers, cell cycle synchronization combined with FACS analysis, of live-cell imaging could provide a more comprehensive analysis of cell cycle progression and potential mitotic defects under these experimental conditions.

Preliminary studies have documented that elevated *SNHG15* levels accelerate tumor adaptation to pharmacological agents while diminishing therapeutic compound effectiveness in malignant cells [16,34]. Our investigation extends current understanding by revealing this long non-coding RNA’s participation in ultraviolet-triggered genomic repair processes, while proposing novel mechanistic insights. There are two plausible explanations for the specific elevation of *SNHG15* post-UV damage observed in our study: (1) cell cycle differences: UV-C irradiation predominantly arrests cells in S/G2 phase, where DNA damage response pathways involving *SNHG15* is heightened. Other DNA damaging agents might induce different arrest profiles or engage distinct checkpoint mechanisms. It is possible that the higher proportion of cells in *SNHG15*-responsive phases post-UV contributes to its elevated levels compared to other damages. (2) Concentration/exposure time: UV dosage, chemical concentration and exposure duration directly influence the magnitude of damage and subsequent transcriptional responses like *SNHG15* upregulation. Differences in the observed *SNHG15* levels could potentially arise if the UV exposure used effectively induced a stronger damage signal than the other agents at the specific doses and timepoints measured. Rigorous dose-dependent curves and time-course analyses for each damage type, calibrated to similar endpoints (like survival fraction), would be essential to rule out concentration/exposure time artifacts. This study demonstrates *SNHG15*’s capacity to orchestrate transcriptional recovery pathways following UV exposure and enhance genomic lesion resolution. Prior studies from multiple research teams have mapped *SNHG15*’s involvement in oncogenic progression through various molecular dialogues, predominantly concentrating on its competitive endogenous RNA (ceRNA) functionality within tripartite RNA networks [35–37]. However, compared to the well-characterized ceRNA paradigm, alternative molecular routes through which *SNHG15* mediates malignant transformation and treatment resistance remain inadequately explored. Our findings propose an expanded functional repertoire for this RNA molecule in cellular adaptation to environmental stressors. Key unresolved questions emerge regarding *SNHG15*’s precise involvement in damage-responsive transcriptional reprogramming, along with its potential coordination with RNA polymerase II complexes arrested at DNA lesions and associated repair machinery. These mechanistic relationships present critical directions for subsequent investigations.

Developing data has identified multiple RNA-associated molecules that form functional complexes with *SNHG15* to influence cellular gene expression patterns. Research demonstrates that thig long non-coding RNA may serve as molecular scaffolds by recruiting epigenetic modifiers like EZH2, thereby facilitating oncogenic growth through transcriptional silencing mechanisms [31].Other studies further reveal *SNHG15*’s ability to stabilize Slug transcriptional regulators via post-transcriptional modifications, significantly enhancing colorectal malignancy development [38]. Our data establish a previously unrecognized regulatory axis between *SNHG15* and CREB5 transcription factors that compromises genomic integrity maintenance in pulmonary malignancies. This molecular partnership demonstrates therapeutic potential as a dual-component intervention point for managing treatment-resistant lung cancers.

In summary, we have elucidated *SNHG15*’s multifaceted contributions to lung cancer progression, bridging gaps between proliferation/migration paradigms and DNA damage response – an underexplored intersection in lncRNA biology. Clinically, these dual functions position *SNHG15* as both a potential therapeutic target and a biomarker of radioresistance in lung cancers. However, current limitations include: (1) the exclusive use of A549 cells, and (2) pending in *vivo* validation. Future studies should investigate *SNHG15* inhibitors in combination with radiotherapy using more clinically relevant samples, like patient-derived xenograft models.

## Materials and methods

### Cell lines and cell culture

MRC-5 primary human lung fibroblast lineage (ATCC, CCL-171), A375 (ATCC, CRL-1619), U2OS (ATCC, HTB-96), MCF-7 (ATCC, HTB-22), IMR-90 (ATCC, CCL-186) were acquired through collaboration with the Francis Crick Institute in London; while the A549 cell line and H1299 cell line derived from pulmonary adenocarcinoma tissue (ATCC, CCL-185) originated from the Shanghai-based Cell Biology Research Center affiliated with the Chinese Academy of Sciences. For STR profiling for cell line authentication, DNA was extracted from all the three cell lines and amplified using multiplex PCR targeting core STR loci, followed by capillary electrophoresis on an ABI 3130xl Genetic Analyzer. STR profiling confirmed more than 80% genotype matches for all three cell lines against reference databases. All cellular models were maintained in Dulbecco’s Modified Eagle Medium enriched with 10% fetal bovine serum, with continuous incubation under standard culture conditions (37°C ambient temperature, 5% CO_2_ concentration).

### Cell treatment

A549 and H1299 cells were seeded in culture dishes and allowed to adhere for 16 hours at 37°C. Medium was replaced with fresh complete medium containing the desired drug concentrations (Cisplatin 5 μM, 5-FU 100 μM, Doxorubicin 1 μM, and DRB 50 μM). Cells were incubated at 37°C with 5% CO_2_ for the desired treatment duration (48 hours for cisplatin, 5-FU and Doxorubicin treatment, 3 hours for DRB treatment).

### Rapid amplification of cloned cDNA ends (RACE)

The 3’ and 5’ rapid amplification of cDNA ends (RACE) analyses were executed employing the SMARTer® dual-terminal amplification system (Takara, 634858). For cellular material preparation, MRC-5 fibroblasts were divided into two experimental groups: baseline samples without treatment and counterparts subjected to 10 J/m^2^ UV-C exposure (recovered for 3 hours). Nucleic acid isolation was completed prior to implementing the terminal extension protocol as delineated in the kit’s technical manual, with amplification reactions being driven by the primers described in S1 Table.

### *In vitro* transcription and translation

The amplified *SNHG15* sequences obtained from MRC-5 cellular RNA underwent coupled transcription-translation processing through the commercial PURExpress^®^ protein expression system (NEB #E6800). Following established protocols provided by the manufacturer, the synthesized polypeptides were subsequently separated via SDS-PAGE. Protein detection was accomplished using sensitive argentometric coloration methodology.

### SNHG15 knockdown experiments

3-5 target-specific 19–21 nt sequences were selected according to Dharmacon’s design tool. Lentiviral vectors pLKO.1 with puromycin resistance were cloned and validated by sequencing. HEK293T cells were transfected with shRNA plasmid with packaging mix. Supernatant was harvested at 72 hours and filtered. A549 or H1299 cells were incubated with viral particles with polybrene (8 μg/mL) for 24 hours at 37°C. Medium was replaced with puromycin addition. After 5 days, the knockdown efficiency was verified by qPCR. The specific oligonucleotide sequences used for *SNHG15* suppression were described in S1 Table.

### Cell synchronization and flow cytometry analysis (FACS)

To achieve cell cycle synchronization at the G1/S boundary using thymidine treatment, we implemented a two-step thymidine inhibition protocol. Cultured cells were initially exposed to 2 mM thymidine (Sigma-Aldrich, T1895) for 14 hours. Following thorough removal of the nucleotide analog through multiple washes, the cells were transferred to fresh culture medium containing 24 μM deoxycytidine (Sigma-Aldrich, D3897) for 9 hours to resume cell cycle progression. Subsequently, a second 14-hour thymidine treatment was applied to restrict cellular populations at S-phase initiation. Complete removal of cell cycle inhibitors was ensured through two cycles of medium replacement prior to experimental time course initiation. Samples were collected at predetermined intervals post-synchronization release for downstream analysis.

For cell cycle profiling, we employed fluorescence-activated cell sorting methodology. Adherent cells grown in 10-cm culture vessels underwent specified treatments before being detached, washed, and processed through fixation and staining procedures. Cellular DNA was labeled using 25 μg/mL propidium iodide (Sigma-Aldrich, 81845) in conjunction with 10 μg/mL RNAse A (Sigma-Aldrich, RNASEA-RO) dissolved in PBS, followed by 30-minute incubation at physiological temperature. Stained cell suspensions were analyzed using a FACSCalibur flow cytometer instrument (BD Biosciences), with data acquisition managed by FACStation software and subsequent processing performed in FlowJo. Each experimental condition maintained a minimum detection threshold of 10,000 cellular events to ensure statistical reliability.

### RNA fluorescence in situ hybridization (FISH)

To visualize *SNHG15* RNA distribution, fluorescence in situ hybridization was conducted on A549 cell cultures grown on sterilized glass substrates using a specialized detection system (RiboBio, C10910). Following standard preparation protocols, cellular specimens underwent sequential processing steps: initial rinsing with PBS preceded fixation using a 3.7% formaldehyde solution supplemented with 10% acetic acid in PBS (pH 7.4), maintained at ambient temperature for 15 minutes. Subsequent membrane permeabilization involved ice-cold PBS containing 0.2%−0.5% Triton X-100 combined with 5 mM vanadyl ribonucleoside mixture (10 mM) (NEB, S1402) for 5 min, followed by three 10-minute PBS washes and a final rinse in 2 x SSC solution (Thermo Fisher Scientific, AM9770). Target-specific DNA probes complementary to SNHG15 sequences (RiboBio) were applied overnight (12–16 hr) at 37°C under light-protected conditions using humidity-controlled chambers optimized for monolayer cell cultures. Post-hybridization processing included nuclear counterstaining with DAPI. Fluorescence signal documentation was achieved using a high-resolution confocal imaging system (Leica, TCS SP8) equipped with laser scanning capabilities.

### Clonogenic assay

Following enzymatic dissociation using Trypsin-EDTA solution, *SNHG15*-deficient A549 cell populations were quantified and plated in 6-well culture dishes at sparse concentrations (100–200 cells/well) to assess clonal expansion capability. Cellular clusters developing during the 14–21 day incubation phase underwent fixation and staining with 0.5% crystal violet dye solution. Aggregates were subsequently counted.

### Xenograft formation

In total, 24 male BALB/c nude mice (6-weeks-old) were acquired from Vital River Laboratories (Beijing, China) and housed in sterile environments. Male mice are the preferred model for xenograft tumor transplantation (especially with A549 cell line which exhibits relatively slow subcutaneous tumor formation in nude mice) due to their complete immunodeficiency and stable physiological state. The core rationale lies in circumventing the potential immune rejection risk associated with female mice. The nude mice were randomly divided into normal group (wild-type A549 or H1299 cell injection, n = 12) and model group (SNHG15-KD A549 or H1299 cell injection, n = 12). Bilateral flank injections containing A549 or H1299 cell suspensions (1 x 10^8^ cells/ml) were administered subcutaneously, using cell lines genetically modified to stably express either shSNHG15 or control shRNA constructs. Tumor progression was monitored triweekly, with volumetric assessments employing the modified ellipsoid formula: V = 0.5 x length x width^2^. Following a 28-day observation period, euthanasia was performed through CO_2_ asphyxiation followed by confirmatory cervical dislocation, with subsequent photographic documentation of excised neoplastic tissues. Experimental cohorts consisted of six animals per genetic modification group.

This research protocol received formal approval from the Institutional Animal Ethics Committee of Guangzhou Medical University (Approval No. GY2023–193). All specimens were accommodated in controlled biocontainment units maintaining temperatures between 19–23°C, with standardized photoperiod regulation (12-hour light cycles from 07:00–19:00 daily) and independent air filtration systems.

### Wound healing assay

To evaluate the mobility of A549 and H1299 cells, a scratch-based migration analysis was performed. Cellular samples were initially seeded in 6-well culture dishes containing serum-depleted medium for half a day. Subsequently, a linear scratch was mechanically introduced in the monolayer using a sterile pipette tip. Following the prescribed incubation interval [39], cellular movement patterns at the scratch interface were photographically documented using an inverted optical imaging system (40 x magnification, model CKX41F, Olympus, Tokyo, Japan).

### Immunofluorescence

A549 and H1299 cell cultures grown on glass coverslips were divided into experimental groups receiving UV-C irradiation (10 J/m^2^) or serving as untreated controls. Following irradiation, cellular recovery proceeded for designated time intervals. Samples underwent chemical fixation using 4% paraformaldehyde solution (15 min exposure) followed by membrane permeabilization with PBS-T (PBS plus 0.3% (v/v) Triton X-100). For CPD detection, nucleic acid denaturation was achieved through 5-minute hydrochloric acid treatment (2 M concentration). Subsequent nonspecific binding prevention involved immersion in a blocking medium containing 10% skimmed milk dissolved in the same detergent solution (PBS plus 0.3% (v/v) Triton X-100). Immunostaining protocols employed three distinct primary reagents: CPD-specific monoclonal antibody (Cosmo Bio, CAC-NM-DND-001), anti-γH2A.X antibody (Abcam, ab22551), or anti-H3S10ph antibody (Abcam, ab5176). All immunoreagents were diluted 1:1000 in blocking buffer and applied overnight under refrigeration conditions. Fluorescent visualization utilized Alexa fluor-488 conjugated secondary antibodies (1 hr incubation at ambient temperature) followed by nuclear counterstaining with DAPI (Vector Laboratories, Inc. Peterborough, UK). Microscopic analysis followed established imaging parameters as referenced in previous methodology.

### Reverse transcriptase quantitative PCR

Total RNA isolation from nascent and mature RNA fractions was conducted with the RNeasy kit (QIAGEN, 74104), following the manufacturer’s guidelines which included column-based DNase I treatment (QIAGEN, 79254). cDNA synthesis was achieved through the PrimeScript™ RT Reagent Kit with integrated genomic DNA elimination (Takara, RR047A). Amplification cycles using iQ SYBR green Supermix (Bio-Rad, 1708880) comprised 30 repetitions of the following thermal profile: 94°C for 15 s (denaturation), 60°C for 15 s (primer annealing), and 72°C for 20 s (elongation). GAPDH-normalized expression levels in experimental groups (e.g., UV-treated samples) were statistically compared to untreated controls unless stated otherwise. Primer sequences for nascent RNA amplification corresponded to those previously reported [40].

### RNA pull-down assay

The SNHG15 segments underwent amplification using primer pairs incorporating T7 and SP6 promoter regions. These amplified sequences served as foundational templates for RNA synthesis employing the T7 transcription system combined with biotin labeling reagents (Roche, 11685597910). Synthesized RNA products underwent DNase I digestion (Qiagen, 74104) under RNase-free conditions followed by purification using silica-membrane columns (Qiagen, 74104). For structural stabilization, 3 μg of biotin-tagged RNA in folding solution (10 mM Tris-HCl pH 7.0, 100 mM KCl, 10 mM MgCl_2_) underwent thermal cycling: initial denaturation at 95°C (2 min), rapid chilling on ice (3 min), and structural equilibration at ambient temperature (30 min). The folded RNA was combined with either chromatin fractions or cytoplasmic lysates (MRC5_va lineage, ~ 1 mg total protein) in 500 μl binding solution for 60-minute room temperature incubation.

Magnetic streptavidin particles (Invitrogen, 65001; 50 μl suspension) were introduced to each mixture for additional 60-minute binding. Subsequent processing involved five rapid washes with binding buffer followed by protein elution using denaturing buffer. Eluted components underwent immunoblot analysis or were separated through density-based electrophoresis for subsequent spectrometric characterization.

### RNA immunoprecipitation combined with quantitative PCR

A549 cell suspensions were seeded into 15-cm culture vessels at 5 x 10^6^ cells per container and exposed to UV-C irradiation (10 J/m^2^). Following a 3-hour incubation period, RNA-protein interaction analyses were performed using modified protocols. The celluar disruption solution contained dual enzymatic inhibitors: SUPERase-In RNase inhibitor (1000 U/ml, Ambion, AM2694) and protease inhibitor, while the purification buffer was supplemented with vanadyl ribonucleoside compounds (10 mM, NEB, S1402). Antibodies targeting CREB5 protein (Abcam, ab168928) were employed during precipitation procedures. Both isolated RNA fractions from immunoprecipitation and unprocessed RNA samples underwent quantitative reverse transcription analysis using SYBR Green fluorescent detection reagents (Bio-Rad, 1708880). Oligonucleotides specific for *SNHG15* transcript amplification were described in S1 Table.

### Quantification and statistical analysis

Statistical significance was evaluated using a two-tailed Student’s *t*-Test, where a probability value below 0.05 denoted significance.

## Supporting information

S1 Fig Characterization of *SNHG15*, Related to Fig 1. (A) Semi-quantitative analysis of transcript isoform abundance for *SNHG15* pre- and post-UV irradiation derived from A549 cells. Band intensity corresponding to the two variants of *SNHG15* was assessed via agarose gel electrophoresis and subsequently quantified. Data normalization was conducted using 18S rRNA as the internal reference control. Error bars represent means ± S.D. from three independent experiments. **p < 0.01, and ***p < 0.001 (Student’s *t*-Test). (B) Semi-quantitative analysis of transcript isoform abundance for *SNHG15* in distinct cancer cell lines. Band intensity corresponding to the two variants of *SNHG15* was assessed via agarose gel electrophoresis and subsequently quantified. Data normalization was conducted using expression levels in MRC-5 cells as the internal reference control. Error bars represent means ± S.D. from three independent experiments. (C) Silver-stained peptide gel of in *vitro* translation products derived from the *SNHG15* construct. In *vitro* translation was performed using a rabbit reticulocyte lysate system programmed with (+) or without (-) in *vitro* transcribed *SNHG15* mRNA. The samples in the last two lanes are a 100-fold dilution of the samples in the first two lanes. (D) (*top panel*) the IC50 concentrations for cisplatin, 5-FU and doxorubicin detected in A549 cell line. (*bottom panel*) qRT-PCR analysis of *SNHG15* expression in human lung fibroblasts treated using the IC50 concentrations of the indicated drugs (for 24 h) or UV-C irradiation (recovered for 24 h). Error bars represent means ± S.D. from three independent experiments. *p < 0.05, and **p < 0.01 (Student’s t-test).(E) qRT-PCR analysis of *SNHG15* expression in various human cell lines treated with UV-C irradiation (recovered for 24 h). Error bars represent means ± S.D. from three independent experiments. *p < 0.05, and **p < 0.01 (Student’s t-test).
(TIF)

S2 Fig Depletion of *SNHG15* inhibits colony formation and cell migration in A549 cells, Related to Fig 2. (A) (*left panel*) Integrative Genomics Viewer (IGV) visualization of RNA sequencing data depicting SNHG15 expression patterns in human lung fibroblast MRC-5 cells synchronized and released from G1/S phase. The genomic architecture of the *SNHG15* locus is schematically represented above the tracks. (*right panel*) Flow cytometry analysis of cell cycle distribution at specified time points post-release. (B) RNA fluorescence in situ hybridization (FISH) was performed to examine the spatial distribution and expression dynamics of the lncRNA *SNHG15* in A549 human lung adenocarcinoma cells under asynchronous growth conditions and following synchronization at the G2/M phase boundary. Scale bar = 50 µm. (C) (*left panel*) Quantitative PCR Validation of *SNHG15* knockdown efficiency in cells transfected with two distinct shRNAs targeting independent sequences of the lncRNA. Error bars represent means ± S.D. from three biologically independent experiments. Statistical significance was determined by two-tailed Student’s t-test (*p < 0.05, **p < 0.01). A schematic illustrates the target regions of the two shRNA constructs. (*right panel*) Semi-quantitative analysis of transcript isoform abundance for *SNHG15* derived from UV-C irradiated A549 or H1299 cells. Band intensity corresponding to the two variants of *SNHG15* was assessed via agarose gel electrophoresis and subsequently quantified. Data normalization was conducted using shCtrl cells as the internal reference control. Error bars represent means ± S.D. from three independent experiments. *p < 0.05, and **p < 0.01 (Student’s *t*-Test). (D) Cell cycle distribution. Quantitative analysis of cell cycle progression in wild-type and *SNHG15*-depleted H1299 cells revealed significant perturbations upon *SNHG15* knockdown. All bars represent means ± S.D. from three biologically independent experiments normalized to account for potential plate-to-plate variability. Statistical analysis for differences in cell populations in each phase compared to control. *p < 0.05, and **p < 0.01 (Student’s *t*-Test). (E) Relative proliferation rates were determined using MTT proliferation assay in H1299 cells. Results were normalized to day 1. Mean values of three independent experiments ± S.D are presented. Statistical analysis for differences in cell proliferation rates in *SNHG15*-deficient H1299 cells compared to WT. *p < 0.05, and **p < 0.01 (Student’s *t*-Test). (F) (*upper panel*) Representative images of clonogenic assays comparing wild-type A549 cells with *SNHG15*-knockdown counterparts. (*lower panel*) Quantitative analysis of clonogenic capacity, expressed as percentage relative to WT control (set as 100%). Error bars represent means ± S.D. from three biologically independent replicates. **p < 0.01 (Student’s t-test). (A) Top: representative images illustrating the gross morphology of xenograft tumors excised from immunodeficient nude mice following subcutaneous implantation of human H1299 cells. Visual comparison clearly demonstrates that tumors derived from *SNHG15*-KD cells exhibit a substantially reduced size compared to those derived from WT cells. Bottom: Quantification of the tumor volumes in xenograft models. Tumor volumes were measured longitudinally and presented as means ± S.D. (n = 12 mice per group). A statistically significant reduction in tumor volume was observed in the shSNHG15 group compared to the shCtrl group (**p < 0.01, two-tailed Student’s t-test), indicating a potential inhibitory role of *SNHG15* knockdown in tumor progression. (B) Analysis of migration capability in A549 cells before and after knockdown of *SNHG15* by scratch wound healing assay. Knockdown of *SNHG15* significantly impairs the migration capacity of A549 cells. (C) Analysis of migration capability in H1299 cells before and after knockdown of *SNHG15* by scratch wound healing assay. Knockdown of *SNHG15* significantly impairs the migration capacity of A549 cells. (D) Wound closure area quantification data in A549 cells. Error bars represent means ± S.D. from three independent biological replicates. **p < 0.01, ***p < 0.001 (Student’s *t*-Test).
(TIF)

S3 Fig Knockdown of *SNHG15* enhanced genome integrity. (A) (*left panel*) Representative images of immunofluorescence to p-H3S10 (green) in the wild-type and *SNHG15*-depleted A549 cells. Nuclei counterstained with DAPI (blue). Scale bar = 50 μm. (*right panel*) Quantification of percent (%) p-H3S10-positive nuclei for each cell line. A minimum of 500 cells per cell line were analyzed. Each data point is presented as the means ± S.D. from three independent experiments. (B) (*left panel*) DAPI staining of representative fields shows multinucleation and micronuclei in A549 cells. (*right panel*) Quantification of A549 cells with more than two nuclei or micronuclei in each cell line. A minimum of 500 nuclei per cell line were analyzed. Each data point is presented as the means ± S.D., **p < 0.01 (Student’s *t*-Test).
(TIF)

S4 Fig Characterization of *SNHG15* in H1299 cell line. (A) qRT-PCR analysis of *SNHG15* expression in A549 and H1299 cells treated with the indicated doses of UV-C irradiation (recovered for 24 h). Error bars represent means ± S.D. from three independent experiments. *p < 0.05, and **p < 0.01 (Student’s t-test). (B) (*left panel*) representative images of clonogenic survival assay showing *SNHG15*-depleted H1299 cells exposed to varying UV-C doses. (*right panel*) Survival rates (logarithmic scale) as a function of UV-C dose, with error bars representing standard deviation from three independent biological replicates. *p < 0.05, **p < 0.01 versus shCtrl controls (Student’s t-test). (C) Representative immunofluorescence images and quantitative analysis of CPD repair kinetics in wild-type and *SNHG15*-depleted H1299 cells. Cells were stained with anti-CPD antibody (green) at specified time points post-UV irradiation, with DAPI counterstaining (blue) indicating nuclei. Scale bar = 50 μm.(D) Nascent mRNA production in different regions of the human *KIFAP3* gene.H1299 cells were irradiated with 15 J/m^2^ UV-C. Error bars represent means ± SD from three independent experiments. *p < 0.05 compared to the shCtrl cells (Student’s t-test).
(TIF)

S1 File Full sequences of *SNHG15* isoforms. (DOCX)

S1 Table (XLSX)
