# Supplementary figures and images for: The role of lncRNA SNHG15 in UV-induced DNA damage repair
Source: PLoS One. 2025 Oct 9;20(10):e0334414. doi: 10.1371/journal.pone.0334414 (PMC12510568; doi:10.1371/journal.pone.0334414)

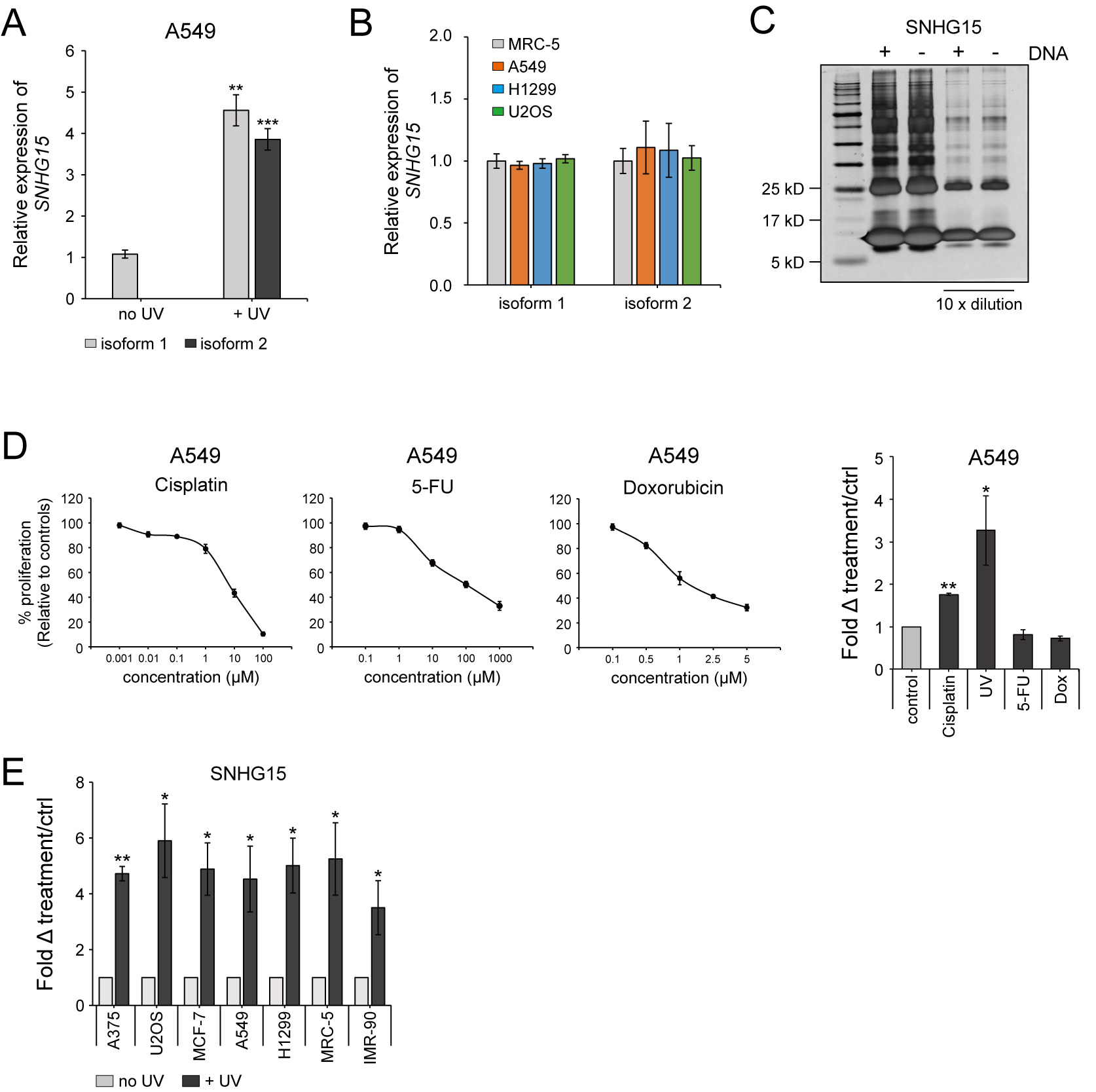

Supplement: S1 Fig — Band intensity corresponding to the two variants of SNHG15 was assessed via agarose gel electrophoresis and subsequently quantified. Data normalization was conducted using 18S rRNA as the internal reference control. Error bars represent means ± S.D. from three independent experiments. **p < 0.01, and ***p < 0.001 (Student’s t-Test). (B) Semi-quantitative analysis of transcript isoform abundance for SNHG15 in distinct cancer cell lines. Band intensity corresponding to the two variants of SNHG15 was assessed via agarose gel electrophoresis and subsequently quantified. Data normalization was conducted using expression levels in MRC-5 cells as the internal reference control. Error bars represent means ± S.D. from three independent experiments. (C) Silver-stained peptide gel of in vitro translation products derived from the SNHG15 construct. In vitro translation was performed using a rabbit reticulocyte lysate system programmed with (+) or without (-) in vitro transcribed SNHG15 mRNA. The samples in the last two lanes are a 100-fold dilution of the samples in the first two lanes. (D) (top panel) the IC50 concentrations for cisplatin, 5-FU and doxorubicin detected in A549 cell line. (bottom panel) qRT-PCR analysis of SNHG15 expression in human lung fibroblasts treated using the IC50 concentrations of the indicated drugs (for 24 h) or UV-C irradiation (recovered for 24 h). Error bars represent means ± S.D. from three independent experiments. *p < 0.05, and **p < 0.01 (Student’s t-test).(E) qRT-PCR analysis of SNHG15 expression in various human cell lines treated with UV-C irradiation (recovered for 24 h). Error bars represent means ± S.D. from three independent experiments. *p < 0.05, and **p < 0.01 (Student’s t-test). (TIF) [file pone.0334414.s001.tif]

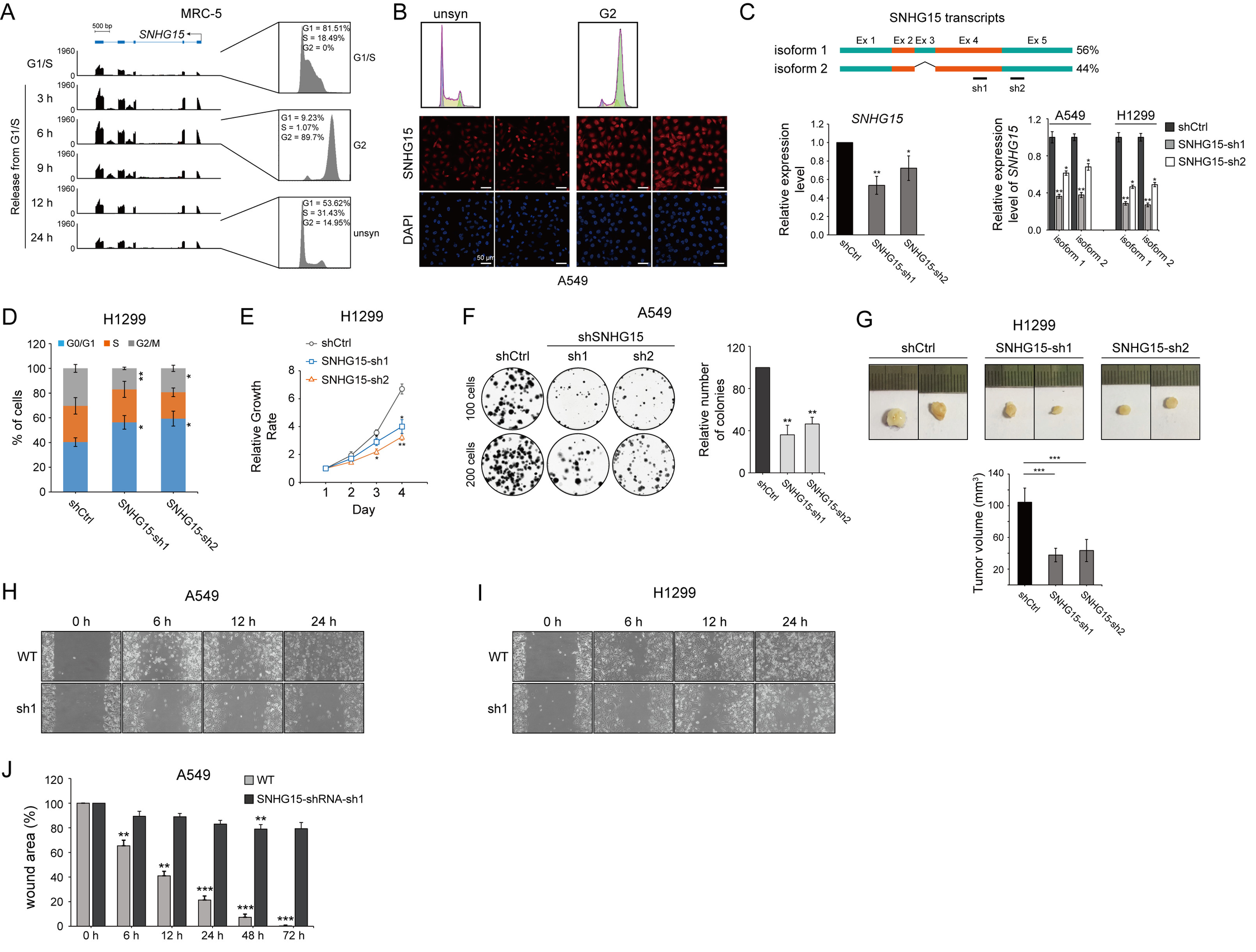

Supplement: S2 Fig — The genomic architecture of the SNHG15 locus is schematically represented above the tracks. (right panel) Flow cytometry analysis of cell cycle distribution at specified time points post-release. (B) RNA fluorescence in situ hybridization (FISH) was performed to examine the spatial distribution and expression dynamics of the lncRNA SNHG15 in A549 human lung adenocarcinoma cells under asynchronous growth conditions and following synchronization at the G2/M phase boundary. Scale bar = 50 µm. (C) (left panel) Quantitative PCR Validation of SNHG15 knockdown efficiency in cells transfected with two distinct shRNAs targeting independent sequences of the lncRNA. Error bars represent means ± S.D. from three biologically independent experiments. Statistical significance was determined by two-tailed Student’s t-test (*p < 0.05, **p < 0.01). A schematic illustrates the target regions of the two shRNA constructs. (right panel) Semi-quantitative analysis of transcript isoform abundance for SNHG15 derived from UV-C irradiated A549 or H1299 cells. Band intensity corresponding to the two variants of SNHG15 was assessed via agarose gel electrophoresis and subsequently quantified. Data normalization was conducted using shCtrl cells as the internal reference control. Error bars represent means ± S.D. from three independent experiments. *p < 0.05, and **p < 0.01 (Student’s t-Test). (D) Cell cycle distribution. Quantitative analysis of cell cycle progression in wild-type and SNHG15-depleted H1299 cells revealed significant perturbations upon SNHG15 knockdown. All bars represent means ± S.D. from three biologically independent experiments normalized to account for potential plate-to-plate variability. Statistical analysis for differences in cell populations in each phase compared to control. *p < 0.05, and **p < 0.01 (Student’s t-Test). (E) Relative proliferation rates were determined using MTT proliferation assay in H1299 cells. Results were normalized to day 1. Mean values of three ind [file pone.0334414.s002.tif]

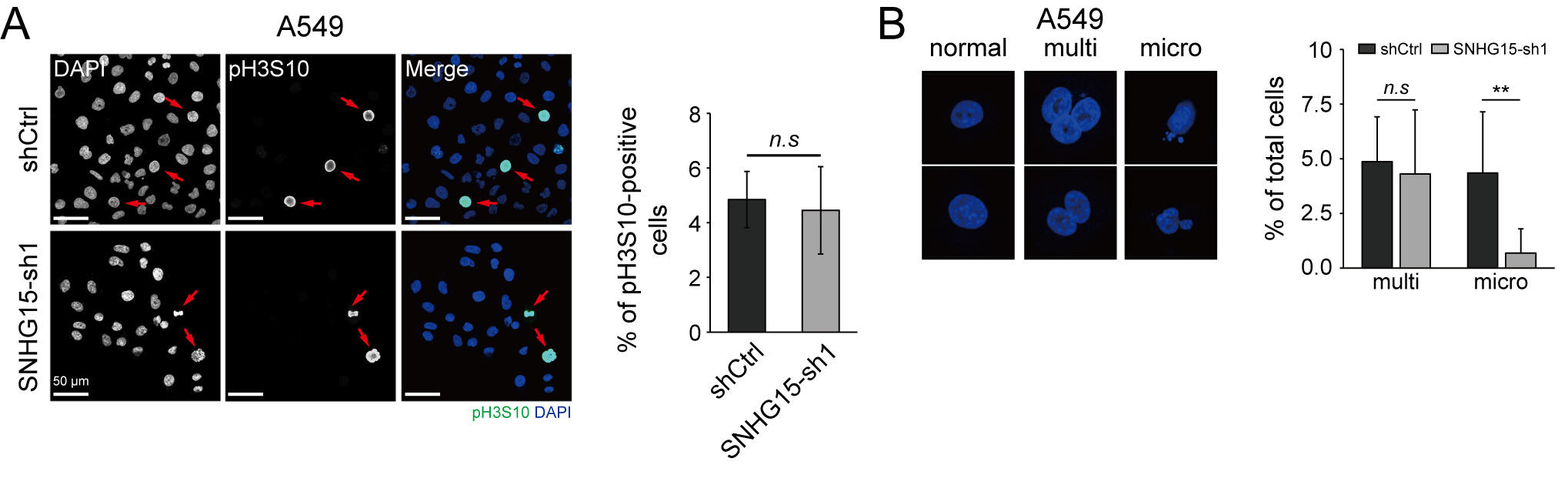

Supplement: S3 Fig — (A) (left panel) Representative images of immunofluorescence to p-H3S10 (green) in the wild-type and SNHG15-depleted A549 cells. Nuclei counterstained with DAPI (blue). Scale bar = 50 μm. (right panel) Quantification of percent (%) p-H3S10-positive nuclei for each cell line. A minimum of 500 cells per cell line were analyzed. Each data point is presented as the means ± S.D. from three independent experiments. (B) (left panel) DAPI staining of representative fields shows multinucleation and micronuclei in A549 cells. (right panel) Quantification of A549 cells with more than two nuclei or micronuclei in each cell line. A minimum of 500 nuclei per cell line were analyzed. Each data point is presented as the means ± S.D., **p < 0.01 (Student’s t-Test). (TIF) [file pone.0334414.s003.tif]

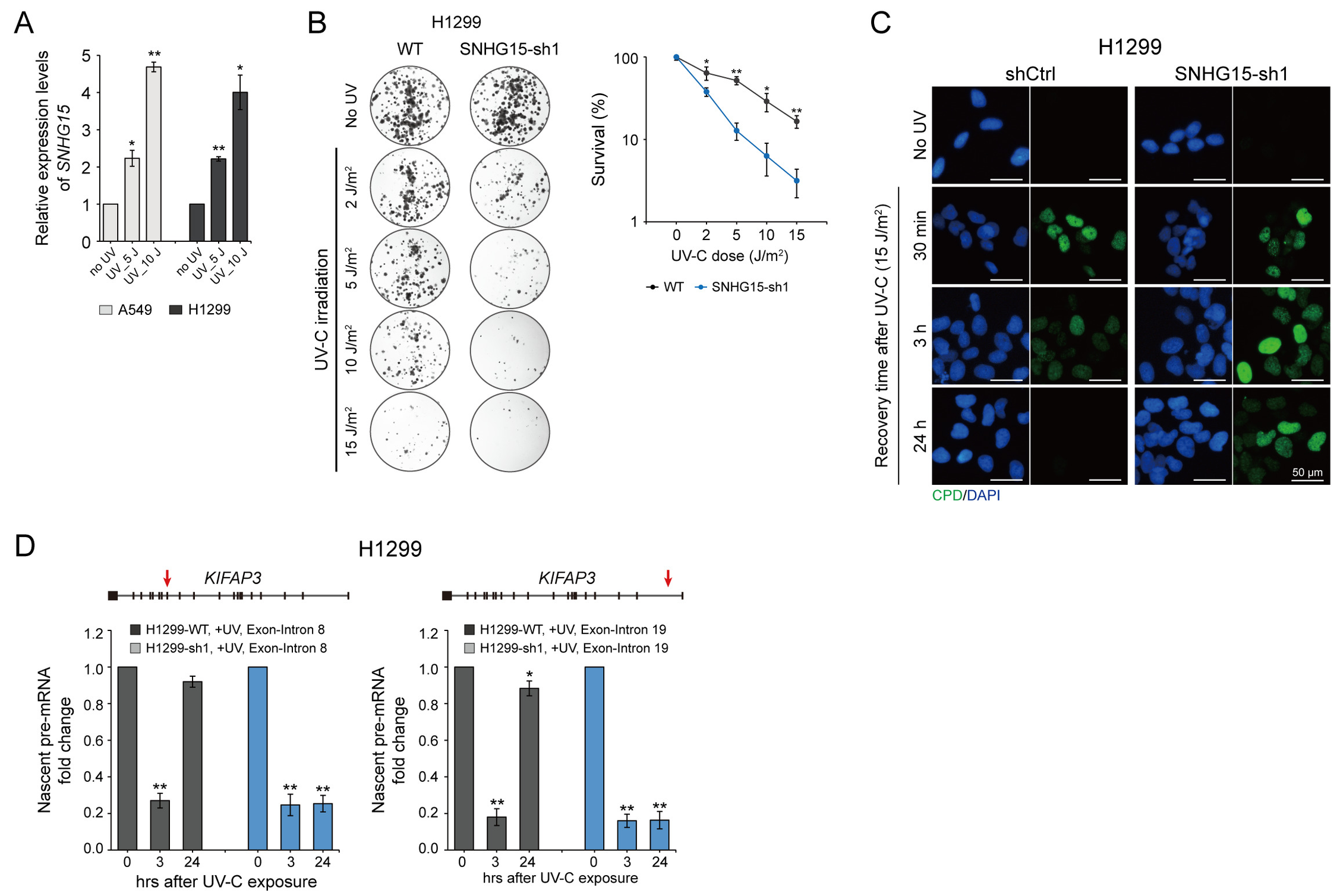

Supplement: S4 Fig — (A) qRT-PCR analysis of SNHG15 expression in A549 and H1299 cells treated with the indicated doses of UV-C irradiation (recovered for 24 h). Error bars represent means ± S.D. from three independent experiments. *p < 0.05, and **p < 0.01 (Student’s t-test). (B) (left panel) representative images of clonogenic survival assay showing SNHG15-depleted H1299 cells exposed to varying UV-C doses. (right panel) Survival rates (logarithmic scale) as a function of UV-C dose, with error bars representing standard deviation from three independent biological replicates. *p < 0.05, **p < 0.01 versus shCtrl controls (Student’s t-test). (C) Representative immunofluorescence images and quantitative analysis of CPD repair kinetics in wild-type and SNHG15-depleted H1299 cells. Cells were stained with anti-CPD antibody (green) at specified time points post-UV irradiation, with DAPI counterstaining (blue) indicating nuclei. Scale bar = 50 μm.(D) Nascent mRNA production in different regions of the human KIFAP3 gene.H1299 cells were irradiated with 15 J/m2 UV-C. Error bars represent means ± SD from three independent experiments. *p < 0.05 compared to the shCtrl cells (Student’s t-test). (TIF) [file pone.0334414.s004.tif]
