# Supplementary material for: The role of lncRNA SNHG15 in UV-induced DNA damage repair
Source: PLoS One. 2025 Oct 9;20(10):e0334414. doi: 10.1371/journal.pone.0334414 (PMC12510568; doi:10.1371/journal.pone.0334414)
Supplement: S1 File — (DOCX) [file pone.0334414.s005.docx]

Full sequence of SNHG15 isoform 1 (long)

GGCGGGACTACGCGGTGACGTCGAGGTGCGCGGCGCAGCGCGCGGCGTCAGTCTTGGCTGGCAGACCTGTACTCCGTACTCCGTACTTCGTAGTCGCAGCGGCGCGGTCTTCGGCAGTCTAGTCATCCACCGCCATCCTGGGCCCCACGTGTTGCCTGACCATTCCTGAGCCCAGGTGGGAGCCGTGGCTGAGGTGACGGTCTCAAAGTGGAAGAGCTTACTGTCACAGCAACTCCTTTGCAAGATGCCCCG**GCCAGGAATAGTTGCTGAACACCCCAGGCCTGCTGAGGTCCCTCCTTGAGTCTCATGTTCAAGCAGTCTT**TGTCCATGAAACTGGGAGGCGACCGTGTTAGCTGCCAGTTCCTGACAGCCACCTCTCACCAGTGGCTTCACTCTGTGTCCCTGACCCAGCACATGGCACAAGAGTGCCTGCCATCCGTCAGTGTTTCTACAGCAGCAATCCCAAGATGCTGGAGCTAGAGGGGACCTGACCTGAGAGAAGATACCTTCAGTGGCTGCCAGGCTGTTCCTTGGAACCTGTGCAGGGATGAGGCCTGCCTGTGTTAATACACCTAGTGAGGAGTGGAGCTGAATTTGAATGCAAGCCTTGGCACCTTAATTGAGCAAGTTTGAAACCTCGCTTGTTGCCCTTCTGGAAGGAGTCAGGAATTTCCAGTTCTGGGCCTGGGCTGTGGGTCTGGCAGACAGACCTCTGGCCCTAGGTTTGGGTGCCAGGTTCTCTGCTTCCAGAATGAGAAGCTTTGCTGTGCACCAGGACCTGGGCC

Full sequence of SNHG15 isoform 2 (short)

GGCGGGACTACGCGGTGACGTCGAGGTGCGCGGCGCAGCGCGCGGCGTCAGTCTTGGCTGGCAGACCTGTACTCCGTACTCCGTACTTCGTAGTCGCAGCGGCGCGGTCTTCGGCAGTCTAGTCATCCACCGCCATCCTGGGCCCCACGTGTTGCCTGACCATTCCTGAGCCCAGGTGGGAGCCGTGGCTGAGGTGACGGTCTCAAAGTGGAAGAGCTTACTGTCACAGCAACTCCTTTGCAAGATGCCCCGTGTCCATGAAACTGGGAGGCGACCGTGTTAGCTGCCAGTTCCTGACAGCCACCTCTCACCAGTGGCTTCACTCTGTGTCCCTGACCCAGCACATGGCACAAGAGTGCCTGCCATCCGTCAGTGTTTCTACAGCAGCAATCCCAAGATGCTGGAGCTAGAGGGGACCTGACCTGAGAGAAGATACCTTCAGTGGCTGCCAGGCTGTTCCTTGGAACCTGTGCAGGGATGAGGCCTGCCTGTGTTAATACACCTAGTGAGGAGTGGAGCTGAATTTGAATGCAAGCCTTGGCACCTTAATTGAGCAAGTTTGAAACCTCGCTTGTTGCCCTTCTGGAAGGAGTCAGGAATTTCCAGTTCTGGGCCTGGGCTGTGGGTCTGGCAGACAGACCTCTGGCCCTAGGTTTGGGTGCCAGGTTCTCTGCTTCCAGAATGAGAAGCTTTGCTGTGCACCAGGACCTGGGCC
